# Spatial movement pattern recognition in soccer based on relative player movements

**DOI:** 10.1371/journal.pone.0227746

**Published:** 2020-01-16

**Authors:** Jasper Beernaerts, Bernard De Baets, Matthieu Lenoir, Nico Van de Weghe

**Affiliations:** 1 CartoGIS, Department of Geography, Ghent University, Ghent, Belgium; 2 KERMIT, Department of Data Analysis and Mathematical Modelling, Ghent University, Ghent, Belgium; 3 Department of Movement and Sports Sciences, Ghent University, Ghent, Belgium; Instituto Politecnico de Viana do Castelo, PORTUGAL

## Abstract

Knowledge of spatial movement patterns in soccer occurring on a regular basis can give a soccer coach, analyst or reporter insights in the playing style or tactics of a group of players or team. Furthermore, it can support a coach to better prepare for a soccer match by analysing (trained) movement patterns of both his own as well as opponent players. We explore the use of the Qualitative Trajectory Calculus (QTC), a spatiotemporal qualitative calculus describing the relative movement between objects, for spatial movement pattern recognition of players movements in soccer. The proposed method allows for the recognition of spatial movement patterns that occur on different parts of the field and/or at different spatial scales. Furthermore, the Levenshtein distance metric supports the recognition of similar movements that occur at different speeds and enables the comparison of movements that have different temporal lengths. We first present the basics of the calculus, and subsequently illustrate its applicability with a real soccer case. To that end, we present a situation where a user chooses the movements of two players during 20 seconds of a real soccer match of a 2016–2017 professional soccer competition as a reference fragment. Following a pattern matching procedure, we describe all other fragments with QTC and calculate their distance with the QTC representation of the reference fragment. The top-*k* most similar fragments of the same match are presented and validated by means of a duo-trio test. The analyses show the potential of QTC for spatial movement pattern recognition in soccer.

## 1. Introduction

In soccer and coaching sciences, the analysis of movement patterns of players has primarily focused on the impact of factors such as field size, number of players [1] on the field and even weather conditions or coach encouragement on the performed movement patterns [2,3]. At a more personal level, the mental impact of the performed movement patterns was studied intensively [4]. More recently, methods have been introduced that combine spatial and contextual information to study performance in soccer [5]. However interesting, these studies fail to detect actual performed spatial movement patterns of players, which are useful for a coach to describe the playing style or tactics of a group of players or of a team. Playing styles in soccer can be described as the general behaviour of a group of players or of a whole team, which is aimed at achieving the defensive and offensive objectives in a match [6]. The impact of different playing styles on team performance can be studied [7, 8] by analysing one or multiple metrics [9] that can be derived from positional data such as, amongst others, ball possession [10, 11], passing directions and distributions [12] or the locations of events such as interceptions, ball losses and set pieces [13]. However, performed playing styles in soccer are influenced by a variety of factors, with even contextual factors such as match status or match venue to be of proven importance [14]. Besides the study on playing styles, neural networks and machine learning techniques have been applied on soccer to detect patterns of tactics based on positional data [15,16]. Detection of reoccurring movement patterns could contribute to the characterization of playing styles and tactics in soccer by describing the movement behaviour of players on the field. In soccer clubs, spatial movement pattern detection is generally done by video inspection and notational analysis [4] of both the own team as well as the opponent. The main aspect of this detection consists of visually finding and annotating similar movement patterns that occur during one or more games, which is a very time-consuming and subjective effort that often has a limited quality due to low observational accuracies of coaches or analysts [17]. Methods for movement pattern detection through the analysis of match events in soccer have been developed in earlier works [18, 19]. However, to the best of our knowledge and as argued by Feuerhake [20], an optimal movement pattern recognition method aimed at the automatic recognition of reoccurring spatial movement patterns of soccer players is currently not available.

When analysing the movements of soccer players during a soccer match, it is of interest to detect spatial movement patterns of one or more players. This means finding different time intervals, in one or multiple games, during which one or more players performed similar movements on the field. This is possible with or without predefining a spatial movement pattern of interest. When no such pattern is used as reference, the analysis can be seen as an example of data mining [20, 21]. It implies that similarities between all possible movements that occur during one or multiple soccer matches are calculated. Possible results include one or multiple groups of movements that have similarities higher than a certain threshold. Although data mining approaches have been used to detect movement patterns in soccer [22, 23, 24], they do not guarantee that the detected movement patterns are meaningful for soccer coaches, analysts or reporters [25, 26]. When a predefined spatial movement pattern is used as a reference, the problem is referred to as pattern matching. Because the dataset is analysed using a reference spatial movement pattern, it is referred to as spatial movement pattern recognition rather than detection. Applied to soccer, pattern matching implies that similarities between the reference movement and all other movements that occur during one or multiple soccer matches are calculated. Consequently, a player movement pattern is recognised when a time interval with a distance lower than a given threshold is found.

In this paper, we investigate the use of the Qualitative Trajectory Calculus (QTC) [27] for the recognition of spatial movement patterns of one or multiple players. Following a pattern matching principle [27], we propose a new method that is aimed at supporting a soccer coach, analyst or reporter to select a specific game fragment as reference and search the database for similar movement patterns for the same or other players. The goal is to allow the user to decide what the pattern of interest is, ensuring that the results have an added value from the sports perspective [26]. We demonstrate this by means of a basic soccer case study. In Section 2, we present a literature overview containing the state of the art in spatial movement pattern recognition both in soccer as well as in other team ball sports. In Section 3, we introduce the proposed method with some basic examples and present the approach and dataset used for the case study. Section 4 presents the results and validation of the case study. Section 5 contains a discussion of the results of this case study and of the proposed method in general. We conclude this paper by presenting options for future research in this field.

## 2. Player spatial movement pattern recognition in sports

Research of performance analysis in sports has seen a huge growth in recent years, as sports data are becoming more widely available due to technological developments, decreasing prices of tracking technologies and sports federations adopting policies for application in official matches [28,29]. Match analysis in soccer originally relied on video images and consisted of quantitative assessments (*e*.*g*. pass frequencies) and qualitative assessments such as expert evaluations [26]. With the current state of the art tracking technology it is possible to log the (*x*,*y*,*t*)-coordinates of soccer players by means of cameras that are mounted around the field [30]. As described by Memmert and Raabe [26], such data allows for physiological and technical assessments (match analysis level 3.0) and dynamical tactical assessments (match analysis level 4.0) of soccer matches. With the trajectories of the players available during the match, the question arises how to analyse the spatial movement patterns performed by the players (match analysis level 4.0), in order to enhance the players’ and team’s performance and, ultimately, win more games. Since spatial movement pattern detection and recognition in various sports uses the same type of data, we first briefly present the state of the art in team ball sports in general before presenting a more focused review of the established methods in soccer.

### 2.1 Team ball sports

In basketball, spatial movement patterns of (multiple) players have been analysed based on the trajectories of players. Space-time movement patterns of both playing dyads as well as whole teams (using a stretch index based on the geometric centre of each team), were studied by Bourbousson *et al*. [31, 32]. Focusing on one aspect of the game, Leite *et al*. [33] studied the effects of defensive pressure on the performed spatial movement patterns. Sha *et al*. [27] introduced the principle of *chalkboarding* in sports analytics, where a user can draw the requested pattern (called ‘a play’) and the system returns the time intervals during which the players performed similar movements. Sha *et al*. use the Euclidean distance between the positions of corresponding players at each timestamp of different plays to calculate the distance between the different plays. As such, identical plays performed on different parts of the field have a relatively high distance from the reference play. Sweeting *et al*. [34] proposed a method for detecting player spatial movement patterns in netball based on a sequence analysis of secondary parameters (derived from the players’ trajectories) used for describing the external load on players.

### 2.2 Soccer

Soccer has attracted substantial research interest in the past years, by academics as well as by private companies. In this paper, however, we focus on a rather small subcategory of sports analytics in soccer, *i*.*e*. spatial movement pattern detection and recognition. For a more general overview of sports analytics in soccer, we refer to the works of Rein and Memmert [30], Sarmento *et al*. [35] and Memmert *et al*. [36].

Most research in spatial movement pattern detection and recognition in soccer makes use of quantitative approaches. Kang *et al*. [37], for example, implemented a method for evaluating the strategic performance of players, based on the regions of the field players consecutively move through during the game. Similar to the team centroid method of Bourbousson *et al*. [31, 32] in basketball, efforts have been made to study spatial movement patterns of a whole team [38, 39], player groups [40] or individual players [41] using the team centroid in soccer. An interface that supports a coach, analyst or reporter at identifying interesting game situations in soccer was created by Shao *et al*. [42]. Other efforts for facilitating the visual abstraction of spatial movement patterns, using clustering methods such as *k*-Means and *k*-Medoids, can be found in Sasha *et al*. [43]. Popular quantitative distance measures for player spatial movement pattern detection and recognition in soccer are, amongst others, the average Euclidean distance [44], the perpendicular and angle distances [45] or the Fréchet distance [46]. Gudmundsson and Wolle [23], for example, use these distance measures for detecting correlations between player movements.

A smaller number of studies are of a qualitative nature, with considerable efforts on the detection of spatial movement patterns through the use of T-patterns [47, 48]. The T-pattern method aims at revealing temporal patterns that are not detectable through classic visualisation techniques. The method was applied to soccer by, amongst others, Sarmento *et al*. [18] and Camerino *et al*. [49] to detect temporal patterns of match events with a spatial component. Feuerhake [20] and Feuerhake and Sester [50] try to discover unknown spatial movement patterns via data mining. At first this was done only for multiple players [50], but later the method was generalized and extended to the trajectories of individual players [20]. The largest difference of this approach with our proposed method (see Section 3) is that in the former, the positions of the players at single timestamps are placed in sequence rather than the movement/displacement during the time intervals between the timestamps. A similar approach was used earlier by Grunz *et al*. [22], who used static team formations and their temporal evolution for spatial movement pattern recognition. Feuerhake later suggested, however, to also use the movement during the time intervals as sequence elements as is done in our method presented below, be it only for single players. Also, using the approach of qualitative description of the movements of multiple players during time intervals, Relative Movement (REMO), introduced by Laube *et al*. [51], can be applied for movement pattern recognition. Laube *et al*. included a soccer example in their paper, but to the best of our knowledge, neither this nor the other methods mentioned above have proven to be sufficient for spatial movement pattern recognition in soccer [20].

## 3. Method

The method we present in this paper is based on the Qualitative Trajectory Calculus (QTC) [52] a calculus that, just as REMO, originated in the field of Geography and was already used for team formation analysis in soccer [53]. In this section, we present the basics of QTC and exemplify its use for spatial movement pattern detection in soccer. Consecutively, we present the case study approach and introduce the used dataset and chosen reference fragment.

### 3.1 The Qualitative Trajectory Calculus for spatial movement pattern recognition in soccer

The Qualitative Trajectory Calculus is a qualitative calculus for describing spatio-temporal relations between two or more Moving Point Objects (MPOs). The most basic form of the calculus, QTC_B_, describes the relative movement of a pair of MPOs during a time interval by means of two QTC-characters. When describing the relative movement of more than two MPOs, pairwise relations are stored in a QTC-matrix. When describing relative movements over multiple time intervals, the consecutive QTC-matrices can be gathered into a sequence.

#### 3.1.1 Proposed method

**Fig 1:**
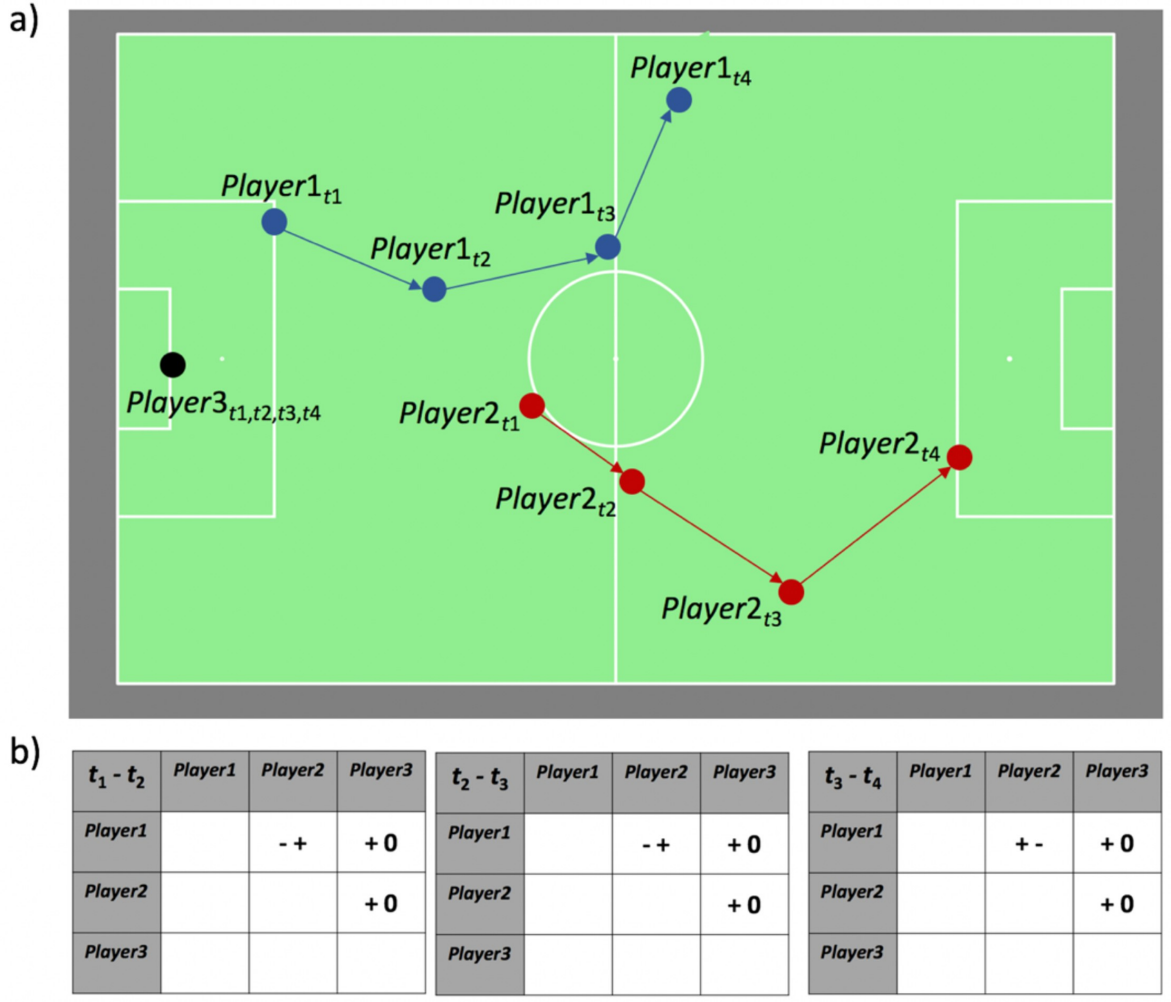
QTC for describing soccer movements. A fragment showing the positions of three players (*Player*1, *Player*2 and *Player*3) at four timestamps (*t*_1_, *t*_2_, *t*_3_, *t*_4_) on a soccer field (a). The QTC_B_-matrix sequence describing the spatial movements during this fragment. In the first matrix of the sequence, the movement of each player from *t*_1_ to *t*_2_ is described with respect to the position of all other players at *t*_*1*_. If this movement is towards another player at *t*_1_, the QTC-relation is denoted by ‘-’, if the movement is away from it, the QTC-relation is denoted by ‘+’. If the movement is neither away from nor towards the marker, the QTC-relation is denoted by ‘0’. The first character in each cell is the QTC-relation of the marker in the row header with respect to the marker in the column header, the second character is the QTC-relation of the marker in the column header with respect to the marker in the row header (b).

By considering soccer players as MPOs, QTC can be used to describe the spatial movements of those players during a particular time interval in a match (Fig 1A). The spatial movements of a set of players during such a well-defined time interval will be referred to as ‘a fragment’ from here on. As such, each fragment can be described by a QTC-matrix sequence (Fig 1B), where the number of players determines the dimension of the individual QTC-matrices that make up the sequence, while the sequence length is defined by the temporal length and resolution of the fragment.

In this case (which is an example of spatial movement pattern recognition), there are two types of fragments. First, there is the reference fragment, which is the fragment of interest to the coach, analyst or reporter, *i*.*e*. for which (s)he wants to find similar fragments in the database. The second type of fragments are the target fragments, which are all other fragments of interest, *e*.*g*. all fragments for the same (or other) players. To start with, the reference fragment is transformed into its QTC-matrix sequence representation. Subsequently, all target fragments are also transformed to their QTC-matrix sequence representations. After that, the distances between the reference fragment and all target fragments are calculated by comparing the QTC-matrix sequences. The comparison of the reference QTC-matrix sequence with a target QTC-matrix sequence starts with the alignment of their matrices [54]. This alignment is similar to the alignment of words, as described by Levenshtein [55], with the difference that a sequence of matrices is aligned instead of a sequence of letters. The cost of aligning two whole QTC-matrix sequences is defined as the sum of the costs of aligning all individual QTC-matrices. If two matrices are identical, they get an alignment cost of zero, and if one or more of the cells of the matrices contain different QTC-characters, a substitution cost is calculated. This substitution cost is based on the conceptual distances between the different QTC-characters [56]. QTC-matrices can be inserted or deleted at the maximum substitution cost. By dividing the calculated distance for every target fragment by the maximum possible distance, relative distances ranging from 0 to 1 are calculated. Target fragments with small distances to the reference fragment are considered to be more similar to the reference fragment than target fragments with larger distances. The result of the spatial movement pattern recognition will thus be a list of the fragments ordered according to the calculated distance. The coach, analyst or reporter can use this list to examine the fragments that contain the movements most similar to the reference fragment (s)he has selected, and can discover whether there is a pattern that occurs regularly either by his/her own team or the opponent team. Ultimately, a coach can use this information to adjust coaching (*i*.*e*. to train specific patterns) to increase the team performance and win more matches.

#### 3.1.2 Difference in speed

**Fig 2:**
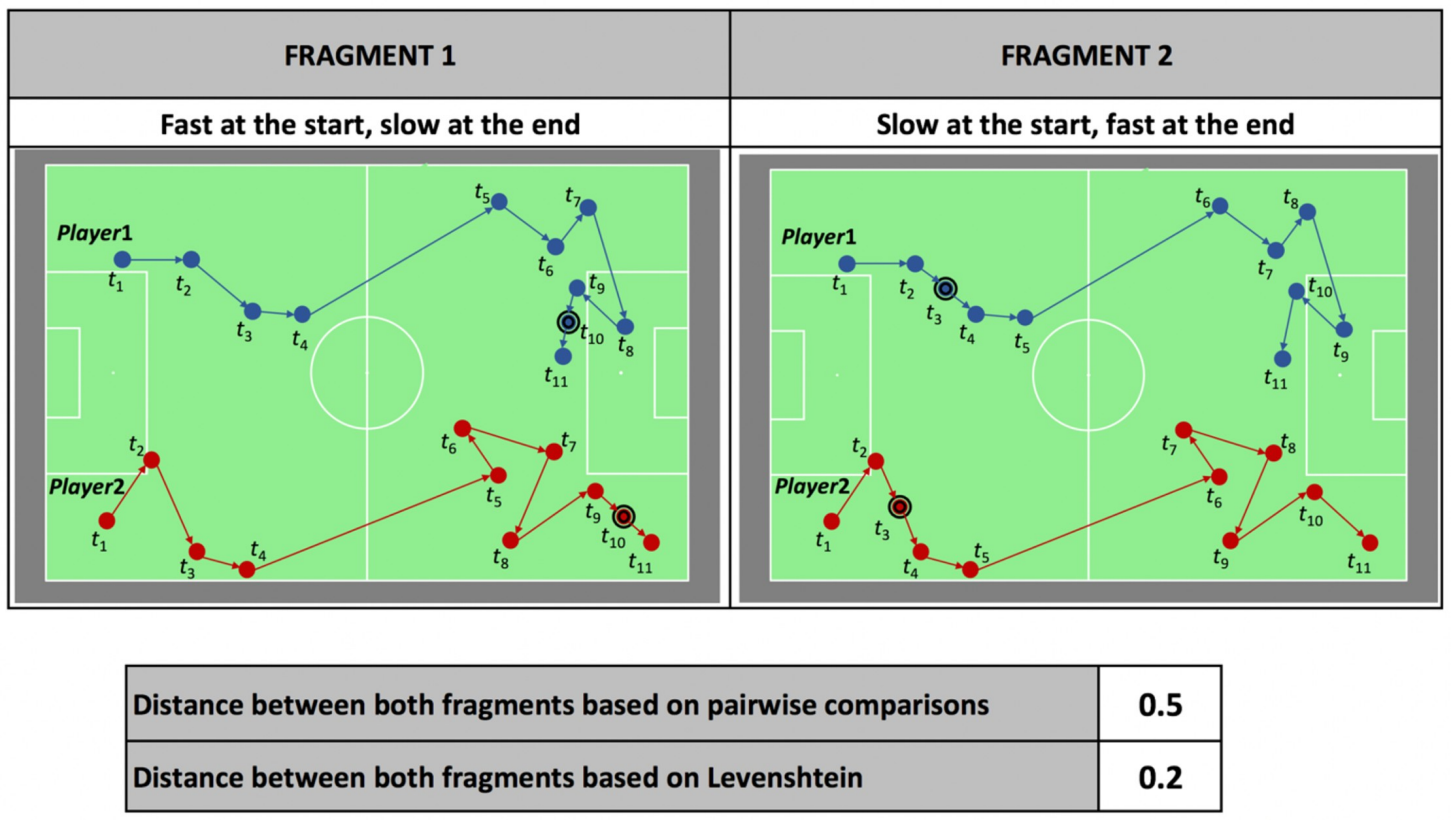
The impact of a difference in speed on the distance between two fragments. Two almost identical fragments with spatial movements of two players (*Player*1 and *Player*2) with small differences in speed along with the distances calculated between them based on pairwise comparisons and on the Levenshtein distance metric. The difference in speed occurs at *t*_10_ and *t*_3_ in fragment 1 and 2 respectively, and is emphasized by a double circle around the players’ positions.

If two fragments contain identical movements, performed at different speeds (*e*.*g*. a higher speed at the beginning and a lower speed at the end, as for fragment 1 in Fig 2), the Levenshtein distance metric [54] will give an appropriate penalty in the distance calculation (Fig 2). As such, the calculated distance between these fragments will not be zero, but will be smaller than the distance that would result from a distance calculation based on pairwise comparisons (where no substitutions or insertions of QTC-matrices are allowed). Fig 2 shows two of such fragments along with the distance between them, computed with both the Levenshtein distance metric as well as with the pairwise-comparison distance calculation. It illustrates the ability of the first method to produce a more suitable, in this specific case lower, distance. Furthermore, by allowing insertions and deletions of QTC-matrices, it is possible to include target fragments with a length different from the reference fragment length. This is an important advantage since in soccer, similar movement actions are often performed at different speeds.

#### 3.1.3 Difference in location on the field

**Fig 3:**
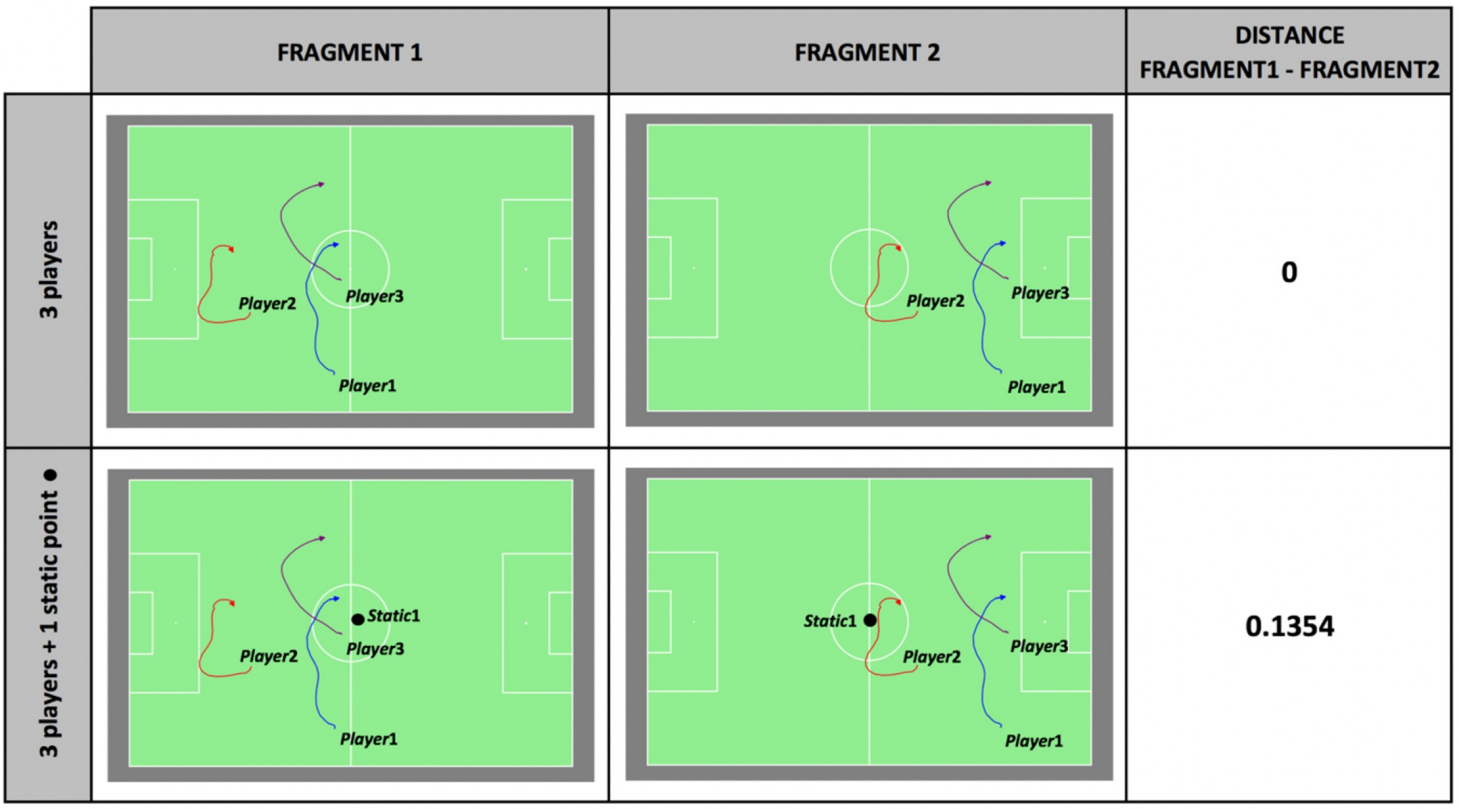
The impact of a difference of location on the field on the distance between two fragments. The impact of a static point (*Static*1) on the distance between two identical fragments with spatial movements of three players (*Player*1, *Player*2 and *Player*3) taking place on different parts of the field.

In order to distinguish between fragments with identical movements taking place on different parts of the soccer field (*e*.*g*. fragment 1 and fragment 2 in Fig 3), static points (*i*.*e*. points that do not move during the fragment) can be added to the QTC-descriptions. In the top part of Fig 3, it is demonstrated that two such fragments, each containing the spatial movements of three players, have a distance of zero because only relations between the different players are described by QTC, which are exactly the same in both fragments. In the bottom part of Fig 3, the middle point of the soccer field is added to the fragment and thus to the QTC-description. Different relations of the players with respect to the static point during both fragments result in a distance value corrected for this difference in location of occurrence on the field.

#### 3.1.4 Current implementation and limitations

The current (non-optimized) implementation of the method was done in the Python programming language. Given the vast number of QTC-matrices that need to be calculated and compared, the number and length of the target fragments are limited. Permutations between players, allowing to recognise spatial movement patterns performed by players other than the ones in the reference fragment, are possible, though require high processing power.

### 3.2 Case study: Dataset and reference fragment selection

The case study consists of a situation where a user (*e*.*g*. soccer coach, analyst or reporter) selects a basic reference fragment from a soccer match and (s)he is presented with the most similar target fragments that occur during the same soccer match.

**Fig 4:**
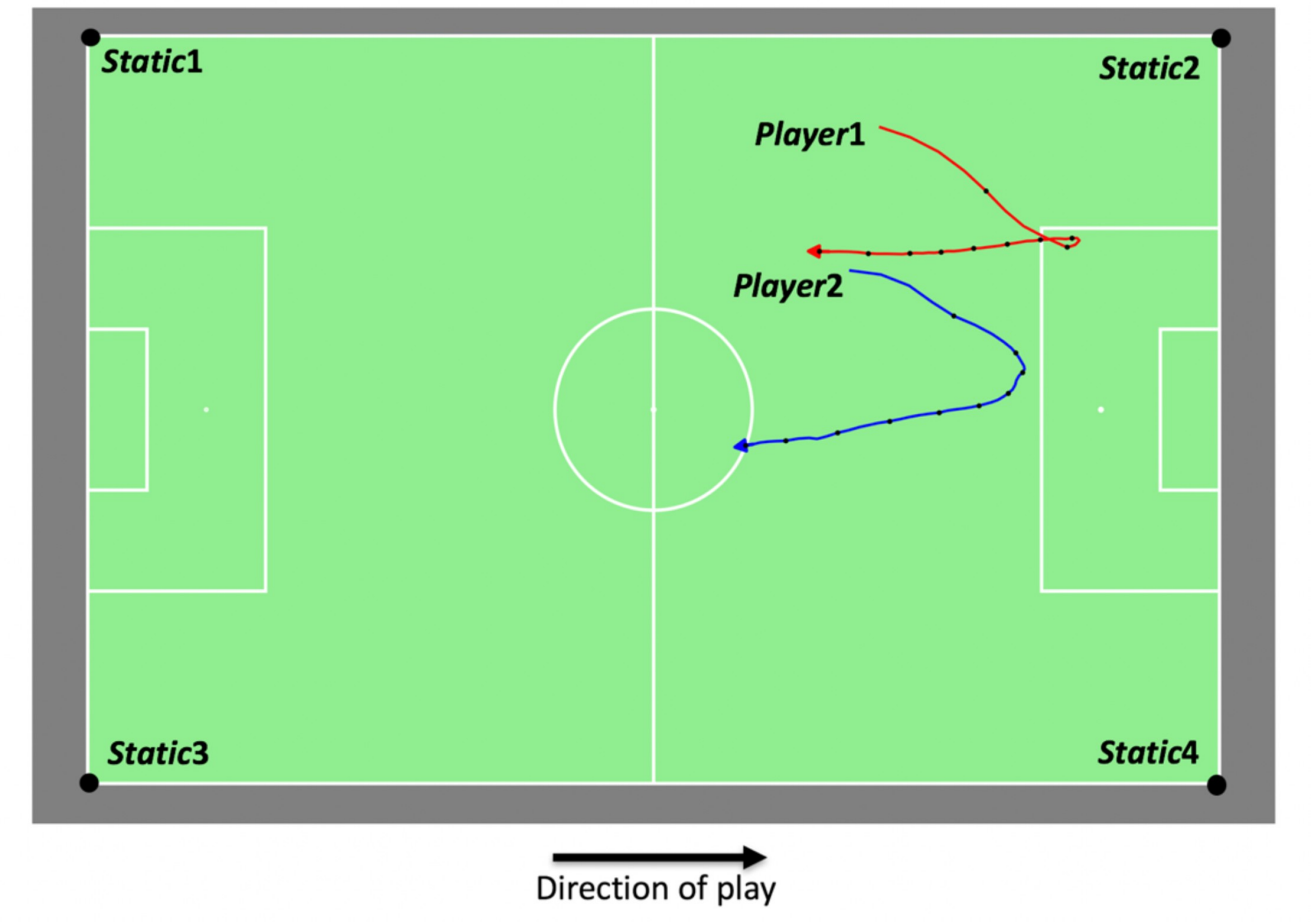
Reference soccer fragment of 20 seconds. This fragment contains the spatial movements of two players (*Player*1 and *Player*2) together with four static points (*Static*1, *Static*2, *Static*3 and *Static*4). Small black dots on the trajectories indicate the positions of the players at every 2 seconds.

The dataset stems from a real soccer match of a 2016–2017 professional soccer competition. Due to privacy concerns, the competition, the teams and the players used in this example are presented anonymised. During the match, players were tracked with a temporal resolution of 25Hz, by a camera-based tracking system with high accuracy, similar to other verified systems [57] used in similar studies [58, 59]. This dataset was collected as the official dataset of the respective competition and contains 144,086 (*x*,*y*,*t*)-coordinates for each of the 22 players on the field. Given the limitations mentioned in Section 3.1.4, we chose a situation where a coach, analyst or reporter selects a rather simple reference fragment consisting of two players. A simple reference fragment is chosen to ensure that relatively similar spatial movements might be found in just one soccer match [20]. Straight sprinting towards the opponents’ goal area, for example, is a regularly trained spatial movement pattern [60] and is in fact the most common pattern to occur before a goal for both the scoring and the assisting player [61]. For this reason, the first occurrence of such a straight run of two players in the match was selected as reference fragment. The 20 seconds (500 timestamps) following the start of the sprint, a common duration for an attacking action in soccer [62], were included in the reference fragment, to add some movement complexity besides the straight run towards the goal. The reference fragment (Fig 4) thus contains the spatial movements of two attacking players of the same team, and starts with them quickly moving towards the opponent’s goal area. Around the sixth second of the fragment, however, the ball possession is lost and the two players start a defensive (parallel) run towards their own goal area. The four corners of the soccer field were added to the fragment as static points, to orientate the movements correctly on the soccer field (see Section 3.1.3). Despite the relative simplicity of this reference fragment, we are convinced that this is a meaningful and interesting situation for a soccer coach, analyst or reporter [60, 61] and an essential starting point to illustrate the method before switching to more complex examples with more players and multiple soccer matches in the future.

For the full length of the match, every 10 timestamps (0.4 seconds) a target fragment of 500 timestamps was created, resulting in a set of 14,359 target fragments. Each target fragment contains the spatial movements of the same players as the reference fragment, during 20 seconds. The temporal resolution of both the reference fragment as well as the target fragments was reduced with a factor 10 to facilitate faster calculations and to reduce the impact of noise in the data on the pattern detection [20]. For each target fragment, the distance between its QTC_B_-matrix representation and the QTC_B_-matrix representation of the reference fragment was computed based on the Levenshtein distance metric, thus allowing for small differences in speed.

## 4. Results

**Fig 5:**
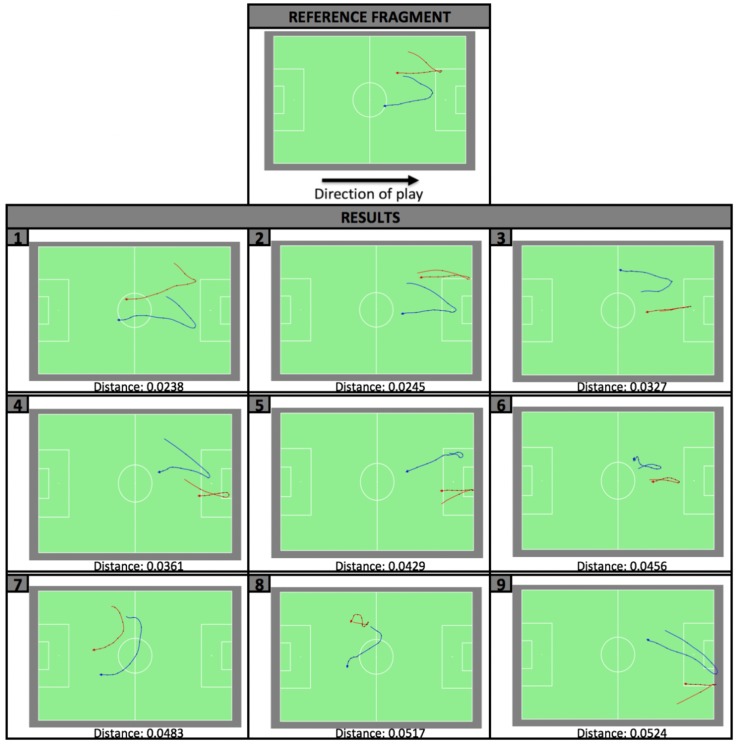
Top nine results of the player spatial movement pattern detection with the reference fragment shown on top. The results are ordered according to ascending distance to the reference fragment, with their rank numbers noted on the left top of the individual visualisations.

Since for each of the target fragments the distance with respect to the reference fragment was calculated, they can be ranked according to this distance. Fragments with a low distance contain movements that are quite similar to the movements in the reference fragment, while the movements in fragments with a high distance will hold little resemblance to the reference fragment. Fig 5 displays the nine most similar, non-overlapping, target fragments ordered according to ascending distance. Non-overlapping fragments are obtained by ordering the fragments according to their rank (starting from rank number 1) and omitting the fragments with higher ranks from the final ranking when they overlapped with a fragment with a lower rank. As such, the number of results was reduced to 221. Depending on the actual spatial movements in the match, a top-*k* (the *k* top ranked target fragments) can be considered as highly similar to the reference fragment (similar to the approach of Sha *et al*. [27]).

### 4.1 Validation of the results

**Fig 6:**
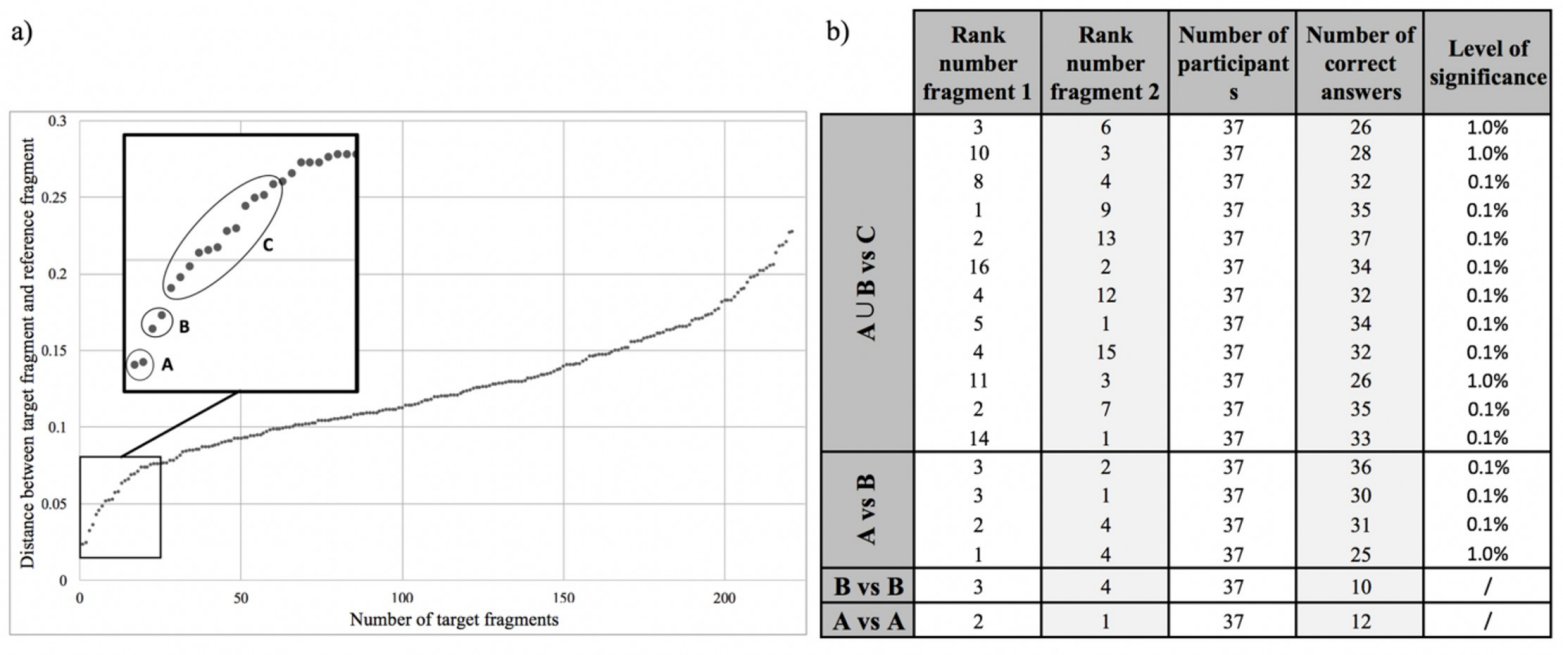
Set-up and results of the duo-trio test. The non-overlapping fragments ordered according to their distance with respect to the reference fragment, and the delineation of the groups A, B and C (a). The questions, answers and significance levels of the duo-trio test (b).

As Feuerhake [20] argues, the verification of spatial movement pattern recognition methods and their results in sports is not a straightforward task due to the lack of a good ground truth. Furthermore, due to the huge variety in different methods, it cannot be assumed that finding the same results as other established methods is desirable nor that it should be the goal. As such, conform to other studies in this field, validation was firstly done by visual comparison of the top-*k* results with the reference fragment. After successfully validating the results in this manner, we proceeded by statistically testing the ranking of the top-*k* results presented in Fig 5. To that end, we ordered the non-overlapping target fragments according to their distance with the reference fragment and detected two distinct groups of fragments (groups A and B in Fig 6A), containing the fragments with rank numbers 1 & 2 and 3 & 4, respectively. Considering the regularly increasing distance curve for fragments with rank numbers above 4, we created a control group C containing 12 elements (see Fig 6A). For validation, we wanted to test whether a test panel would confirm the ranking in Fig 5. Our test panel consisted of 37 bachelor students of the Department of Movement and Sports Sciences of Ghent University with a good knowledge of soccer. In their curriculum they have had at least two years of soccer classes, including practical sessions and theory on technique and tactics in soccer. The study received institutional approval (Ghent University) and the participants’ informed consent was obtained (verbally) and witnessed by the academic teacher. As a statistical test, we set up a duo-trio test [63, 64], a statistical test for determining whether a difference exists between two samples, by asking which of the two samples most resemblances a reference fragment. Our duo-trio test consisted of 18 questions (Fig 6B) which were presented to the participants in random order. Each question contained the visualisations (as in Fig 5) of the reference fragment and two sample fragments, of which the participants had to indicate which one was most similar to the reference fragment. Six questions compared the fragments of groups A and B (1 question A vs A, 1 question B vs B and 4 questions A vs B), twelve questions compared the union A∪B with group C. In the latter series of questions, each fragment of C was randomly combined with a fragment of A∪B. Questions for which the participants chose the sample fragment with the lowest rank number as most similar to the reference fragment were considered as correct answers.

The results, aggregated per question, are presented in Fig 6B. The results show that the participants validated the ranking positively, thereby distinguishing between the fragments of groups A∪B and C and between the fragments of groups A and B with high levels of significance [64]. When it came to ordering the elements in groups A and B separately (A vs A and B vs B), the participants did not validate the exact ordering presented in Fig 5. Considering the small differences in distance between the fragments in those groups, especially compared to the distances with the other fragments, we consider this as acceptable. As such, the top-*k* (with *k* = 4) was validated by the duo-trio test [65].

## 5. Discussion

In literature, it is described that, because of the limited accuracy of tracking technologies and the vast number of possible positions of players on the soccer field, it is almost impossible to find two fragments that contain identical spatial movements [20]. With respect to the first issue, it can be noted that QTC is a relative calculus, meaning that only the relative spatial movements in two fragments need to be the same in order to be considered as identical. Small differences in the players’ coordinates of a given spatial movement pattern, for example, do not cause large differences in its QTC-matrix sequence representation. Furthermore, QTC allows for the recognition of spatial movement patterns consisting of identical movements that occur on different places on the field or at different spatial scales. Adding to the qualitative nature of the calculus, both the temporal (as in the case study) as well as the spatial resolution can be reduced to cope with the limited accuracy of the data. The second issue is visible for the results of the case study presented in Section 4, where it is not straightforward to provide a coach, who defined a relatively simple reference fragment, with highly similar fragments of the same match. For the given reference fragment, four highly similar target fragments could be found in the same soccer match (Fig 5). However, when equipped with a big dataset (containing multiple matches or seasons and thus more target fragments) and a clear focus or definition of the pattern of interest, more specific results, and thus better insights, are to be expected.

The advantage of the proposed method is that it can be adapted to the specific needs of the requested analysis. In comparison with similar methods, such as the work of Shao *et al*. [42], the proposed method is characterized by its scale, rotation and translation invariance. This allows to find highly similar spatial movement patterns that occur, for example, on different parts of the soccer field or at a different scale. Different static points could be included, however, to fix orientation and scale. Another advantage is that, while QTC typically describes relations between two or more MPOs, incorporation of static points allows for the comparison of trajectories of single players. Besides this, permutations between players can be included, allowing players to switch roles in target fragments. Furthermore, the Levenshtein distance metric [54] supports the comparison of fragments with different temporal lengths. This means that the target fragments can have a different length than the reference fragment, something that was not included in the case study for reasons of simplicity, and could raise the chance of finding target fragments with lower distances with respect to the reference fragment and thus more similar spatial movement patterns. The advantage of the proposed method is therefore that it allows for the recognition of similar spatial movement patterns that have different temporal lengths or that occur at different speeds.

Moreover, we believe that the proposed method grasps the essence of the way a soccer player positions him/herself on the field during a game. To that end, the player will mostly look at its teammates and opponents, assessing his/her relative position to each of them and adjust his/her location where needed by moving around on the field. This is exactly what QTC describes, *i*.*e*. the changes in relative positions between all of the players during the match. Most other studies, however, such as Sweeting *et al*. [34], only use movements with respect to the field (*e*.*g*. ‘turn 45 degrees on the field’) for the movement pattern detection and recognition, and do not take into account these interactions between players. As such, we believe that the proposed method could support a soccer coach, analyst or reporter to get insight into the spatial movement patterns that occur within one or multiple soccer matches. With the proposed method, one can easily search for a movement pattern, *e*.*g*. to check whether the patterns that were trained were executed during official matches, or get insight in patterns played by opponent teams.

Limitations of the proposed method include the high requirements of computation power and the necessity of recorded coordinates with a fine temporal resolution. Another limitation is that ball possession is not taken into account, which could be solved by adding a postprocessing step. With respect to the high required computation power, we would like to emphasize that possible solutions to reduce the complexity are available, such as the abortion of the Levenshtein distance calculation when the edit distance between two fragments exceeds a predefined threshold. Other solutions might involve the compression of the QTC-representations of the fragments using Low Level (lossless) or even High Level (lossy) compression techniques. Consequently, only the Levenshtein distance between fragments having low distances (below a certain threshold) between their compressed QTC-representations could be calculated.

## 6. Conclusion and future work

In this paper, we introduced a method for the recognition of spatial movement patterns of players movements in soccer. We followed a pattern matching procedure, by searching for target fragments with spatial movements similar to a reference fragment. The basics of the QTC method for spatial movement pattern recognition were introduced and its use was illustrated by a rather simple case study. Tests should be performed on bigger datasets, with more players and more complex spatial movement patterns, with a more rigorous evaluation of the results by real coaches. Furthermore, expanding the approach to the field of data mining, it would be interesting to compare all fragments with each other and thus get insights in frequently played patterns by certain players, thus performing spatial movement pattern detection. These results could then be matched with formations of other teams, *e*.*g*. which players perform specific spatial movement patterns when playing against other specific players. Also, reduction of the complexity and demands for processing power, for which we provided some research suggestions in our paper, should be studied more in depth.
